# Predictive Factors of Type 2 Diabetes Mellitus Remission Following Bariatric Surgery: a Meta-analysis

**DOI:** 10.1007/s11695-014-1391-y

**Published:** 2014-08-08

**Authors:** Guo-Feng Wang, Yong-Xin Yan, Ning Xu, Dong Yin, Yuan Hui, Ji-Ping Zhang, Guan-Jun Han, Ning Ma, Yan Wu, Jing-Zi Xu, Tao Yang

**Affiliations:** 1 Department of Endocrinology Medicine, Lianyungang First People’s Hospital, Affiliated Hospital of Xuzhou Medical College, Tongguan North Road 182, Lianyungang City, Jiangsu Province 222000 China; 2 Department of Gastrointestinal Surgery, Ianyungang First People’s Hospital, Affiliated Hospital of Xuzhou Medical College, Tongguan North Road 182, Lianyungang City, Jiangsu Province 222000 China; 3 Department of Endocrinology Medicine, The Fist Affiliated Hospital of Nanjing Medical University, 300 Guangzhou Road, Nanjing, 210029 China

**Keywords:** Type 2 diabetes, Diabetes remission, Bariatric surgery, Metabolic surgery

## Abstract

**Background:**

Although a few studies have been reported on predictive factors of postoperative diabetes remission, the conclusions remain inconsistent. This meta-analysis aimed to assess the preoperative clinical factors for type 2 diabetes mellitus (T2DM) remission after bariatric surgery.

**Methods:**

The Cochrane Library, PubMed, MEDLINE, Embase, and CINAHL databases were searched. All human studies published in English between 1 January 1992 and 1 September 2013 reporting on the parameters of interest were included.

**Results:**

In total, 15 studies involving 1,753 bariatric surgery patients were selected. Analyses were performed separately for the parameters of interest. T2DM remission was observed to be negatively correlated with age, diabetes duration, insulin use, and HbA1c levels. Baseline body mass index (BMI) and C-peptide levels were positively associated with the remission rate in Asian patients. However, there was no significant association between gender and remission rate.

**Conclusions:**

Patients with younger age, short diabetes duration, better glucose control, and better β cell function were more likely to achieve T2DM remission after bariatric surgery. However, further randomized controlled trials with uniform remission criteria should be conducted to provide more reliable evidence.

## Introduction

It is generally accepted that obese patients with type 2 diabetes mellitus (T2DM) achieve significant and lasting weight loss and T2DM remission after bariatric surgery [1–4]. This remarkable effect can minimize the possibility of future complications, particularly the risk of cardiovascular disease. However, bariatric procedures may be irreversible and associated with considerable short- and long-term risks. Moreover, although most patients achieve a marked improvement in T2DM, not every patient achieves remission after surgery, and in some, substantial improvement is not observed. Optimal outcomes of surgical treatment for diabetes can be obtained only if the most appropriate patients best suited for bariatric surgery are selected. Even more important is the identification of patients who do not respond well to bariatric surgery. Previous studies have identified many preoperative patient factors associated with surgical outcomes, including age, diabetes duration, glycemic control (HbA1c), fasting C-peptide levels, body mass index (BMI), ethnicity, and medications used to manage blood glucose (including oral hypoglycemic agents and insulin). However, the conclusions of these studies are inconsistent. Against this background, the primary aim of this meta-analysis was to identify the preoperative predictors of diabetes remission. After reviewing relevant articles, we focused on the following parameters: age, gender, diabetes duration, insulin use, baseline BMI, and levels of fasting glucose, HbA1c, and C-peptide. Our findings provide important implications for the identification of patients most likely to benefit from bariatric surgery.

## Materials and Methods

### Search Strategy and Article Selection

The Preferred Reporting Items for Systematic Reviews and Meta-Analyses 2009 Guidelines (PRISMA) were used to conduct data extraction [5]. We conducted a comprehensive review of all studies published in the English literature containing the parameters of interest and T2DM remission with any form of bariatric surgery. The Cochrane Library (www.thecochranelibrary.com), PubMed (www.ncbi.nlm.nih.gov/pubmed), MEDLINE (http://www.ncbi.nlm.nih.gov), Embase (http://www.elsevier.com/online-tools/embase), and CINAHL (http://www.ebscohost.com) databases were searched for articles published from 1 January 1992 through 1 September 2013, with the following search terms: “diabetes* [All Fields],” “surg* [All Fields],” “operat* [All Fields],” (“diabetes mellitus” [MeSH Terms] OR “diabetes” [All Fields] AND “mellitus” [All Fields]) OR “diabetes mellitus” [All Fields] OR “diabetes” [All Fields] and “remission* [All Fields],” or “improve* [All Fields].” Further searches were performed by reviewing abstract booklets and review articles. Relevant articles referenced in these publications were downloaded from the databases and used to widen the search results.

### Selection of Studies

Two reviewers (G.F.W. and Y.X.Y.) independently screened the retrieved articles. All clinical studies reporting on the parameters of interest and T2DM remission were selected and assessed independently and then cross-checked. T2DM remission was defined as cessation of glucose-lowering medications, and it was grouped according to specific HbA1c thresholds. There were no restrictions regarding the surgical procedure. All qualifying articles were then reviewed to verify that each contained sufficient data for analysis, including continuous data reported as means with standard deviations and sample size and dichotomous data reported as a number with sample size. Case reports, conference abstracts, review articles, and non-human studies were excluded. Studies with the same or overlapping cohort of patients were identified as “kinned,” and the largest dataset was chosen from those studies and counted only once.

### Data Extraction

Two reviewers (Y.X.Y. and G.F.W.) independently extracted data, and disagreements were resolved through discussion before analysis. Any unresolved disagreement was judged by the third investigator. Details extracted from the studies included the following: first author, publication year, country, ethnicity, study setting, time frame, study design, sample size, patient age, gender composition, duration of T2DM history, baseline BMI, fasting glucose levels, C-peptide and HbA1c levels, insulin use, type of surgical procedure performed, and definition used for remission. We did not contact authors for unreported data.

### Assessment of Methodological Quality

Two reviewers (Y.X.Y. and G.F.W.) independently assessed the quality risk of bias in each included study using the Newcastle–Ottawa Scale, which was developed to assess the quality of non-randomized studies [6]. The scale awards a maximum of nine points. Studies of high quality received a score of the maximum nine points, studies of medium quality scored seven to eight points, and studies of low quality scored less than seven points [7].

### Statistical Analysis

RevMan 5.0 software (Cochrane Collaboration; http://www.cochrane.org/) was used for statistical analysis of the data. We summarized the available data from all trials reporting results. For results of continuous outcomes, weighted mean differences (WMDs) and standard deviations were assessed. However, for dichotomous outcomes, risk ratios were used to calculate the case results using odds ratios (ORs) and 95 % confidence intervals (CIs). Significant heterogeneity was indicated by a probability (*P*) value of <0.1 or heterogeneity (*H* ^2^) value of >50 %. A random effects model was used if there was a significant heterogeneity; if not, a fixed effects model was used. For heterogeneous studies in the meta-analysis, the results were cautiously interpreted. Publication bias was evaluated by constructing funnel plots with visual assessment of asymmetry. Using the “trim and fill” method to identify and correct for funnel plot asymmetry arising from publication bias, pooled effect estimates were recalculated for each analysis.

## Results

### Characteristics of Eligible Studies

**Fig. 1:**
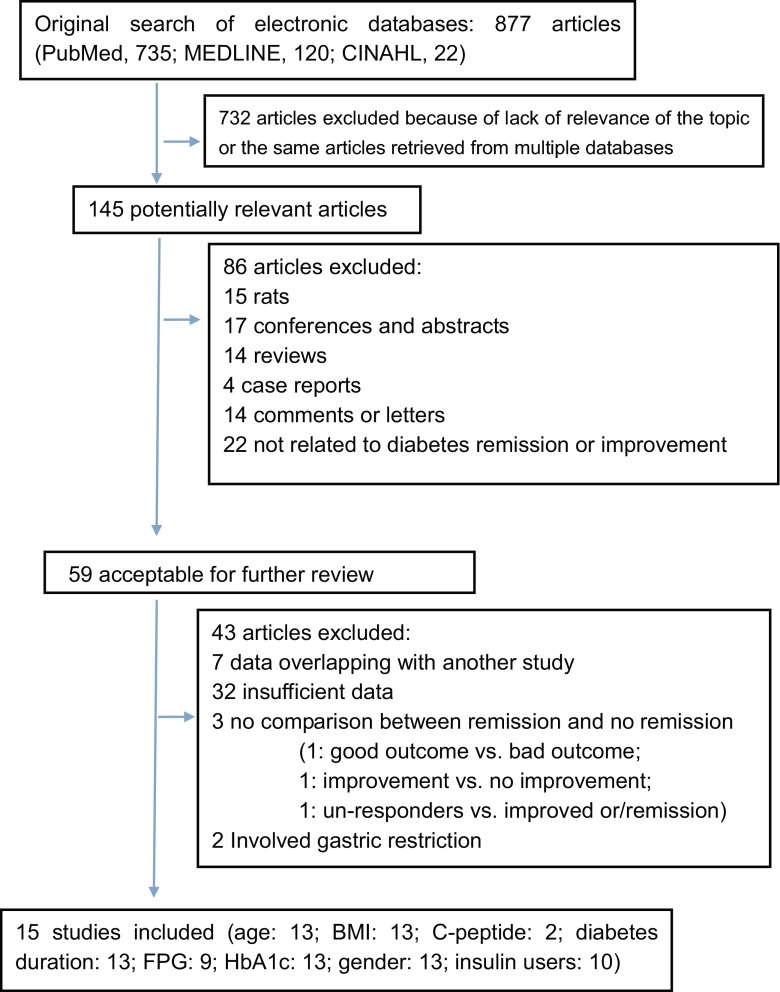
Selection of articles for inclusion in meta-analysis

A total of 877 publications were identified through searching the literature databases and cited references. Of these, 732 were excluded because of the lack of relevance to diabetes remission or improvement. After further evaluation, we excluded an additional 128 studies because of insufficient data, abstract only, review article, or overlapping data with another study. Finally, 15 studies were selected. A flow diagram outlining the systematic review process is presented in Fig. 1.

The 15 eligible articles included a total of 1,753 T2DM patients [8–22]. Most studies were conducted in the USA (seven), with the others conducted in Brazil (one), Taiwan (one), Italy (one), Korea (one), China (one), Spain (two), and New Zealand (one). Of these 15 studies, nine were retrospective, five were prospective, and one was an observational study. The study participants generally had poor glycemic control. Laparoscopic Roux-en-Y gastric bypass (LRYGB) was performed in 4 studies, RYGB was performed in 10 studies, and laparoscopic sleeve gastrectomy (LSG) was performed in 2 study. The follow-up interval ranged from 6 to 36 months, with the most frequently reported interval being 12 months. The setting for the studies was most commonly an academic hospital, with one study each in a community hospital and private practice. The majority of the included studies were considered of good quality by our assessment criteria (score of ≥7). The characteristics of the included articles are presented in Table 1.

**Table 1:** General characteristics of the studies included in the meta-analysis

Author	Year	Country	Surgery procedure	Date of surgery	Setting	Time frame	Sample size	T2DM remission criterion		NOS	Follow-up (months)
								HbA1c (%)	Fasting glucose levels (mg/dl)		
Kadera et al.	2009	USA	RYGB	2000–2006	University hospital	Retrospective	71	<7 %	Unknown	7	≥12
Huang et al.	2011	Taiwan	LRYGB	December 2008–January 2010	Hospital	Prospective	22	<6	<100	8	12
Hirsch et al.	2012	Brazil	RYGB	January 2001–January 2006	University hospital	Retrospective	28	<6	<100	9	≥24
Kim et al.	2011	Korea	RYGB	July 2009–December 2009	Hospital	Prospective	50	<6.5	<126	8	≥12
Levi et al.	2013	Spain	LRYGB (52), BPD (72), LSG (17)	2006–2011	Hospital	Retrospective	141	<6	<100	9	12
Schauer et al.	2003	USA	LRYGBP	July 1997–May 2002	University hospital	Retrospective	191	5–6	≤110	8	19.5 (6–54)
Nannipieri et al.	2011	Italy	RYGB	Unknown	University hospital	Prospective	32	<6.5	<126	8	12
Hayes et al.	2011	New Zealand	RYGB	November 1997–May 2007	University hospital	Prospective	130	<6	<126 %	9	≥12
Kim et al.	2010	USA	RYGBP	May 2002–December 2006	University hospital	Retrospective	219	<7	Unknown	8	≥12 (26.4 ± 12.8)
DiGiorgi et al.	2010	USA	RYGB	Unknown	A single institution	Retrospective	42	<6	<124	8	≥36
Torquati et al.	2005	USA	RYGB	Unknown	University hospital	Prospective	79	Normal	Normal	7	12
Yan	2013	China	RYGB	March 2009–March 2011	General hospital	Retrospective	99	<6.5	<126	9	12
Jimenez A et al.	2013	Spain	RYGB	Unknown	University hospital	Observational	18	<6.5	<126	7	≥24
Blackstone et al.	2012	USA	RYGB	2001–2008	Community hospital	Retrospective	505	<5.7	<100	8	14
Biro et al.	2013	USA	LRYGB, LAGB, LSG	August 2006–February 2011	University hospital	Retrospective	39	<6	<100	6	12

*RYGB* Roux-en-Y gastric bypass, *LRYGB* laparoscopic Roux-en-Y gastric bypass, *LRYGBP* laparoscopic Roux-en-Y gastric bypass procedure, *BPD* biliopancreatic diversion, *LSG* laparoscopic sleeve gastrectomy, *LAGB* laparoscopic adjustable gastric banding, *HbA1c* glycosylated hemoglobin, *NOS* Newcastle–Ottawa Scale

### Patient Characteristics

The mean patient age at baseline varied among the studies and ranged from 33 to 54 years. The mean BMI at baseline ranged from 26.9 to 51.6 kg/m^2^. Resolution of T2DM was defined and reported in a variety of ways (HbA1c levels of <5–6, 5.7, 6.5, and 7.0 %) without glucose-lowering therapy. The patient characteristics of the included articles are presented in Table 2.

**Table 2:** Baseline variables of the included studies

Author	Sex (male/female)		Age (years)		BMI (kg/m^2^)		Duration (years)		Fasting glucose levels (mg/dl)		C-peptide (ng/ml)		HbA1c (%)		Insulin use (*n*)	
	Remission	No remission	Remission	No remission	Remission	No remission	Remission	No remission	Remission	No remission	Remission	No remission	Remission	No remission	Remission	No remission
Kadera et al.	21/14	27/9	46.0 ± 10.1	48.4 ± 8.9	48.7 ± 7.9	49.4 ± 6.8	7.2 ± 6.8	11.0 ± 6.6	Unknown	Unknown	Unknown	Unknown	8.2 ± 1.8	8.5 ± 1.6	35	36
Huang et al.	2/11	0/9	41.69 ± 10.77	55.56 ± 5.028	32.37 ± 2.57	28.06 ± 2.30	3.08 ± 2.71	11.22 ± 6.91	192.92 ± 81.47	231.22 ± 94.56	Unknown	Unknown	9.02 ± 2.10	9.52 ± 1.95	2	2
Hirsch et al.	1/14	2/11	55.7 ± 1.2	52.6 ± 2.3	46.5 ± 2.2	44.8 ± 7.8	8 ± 1.72	10.62 ± 1.78	Unknown	Unknown	Unknown	Unknown	7.9 ± 1.0	7.5 ± 0.8	7	8
Kim et al.	10/24	6/10	43.2 ± 10.3	44.6 ± 10.9	34.5 ± 2.6	34.5 ± 2.7	4.3 ± 3.4	7.4 ± 3.9	190.2 ± 43.3	205.5 ± 69.6	3.81 ± 1.98	3.33 ± 1.21	8.28 ± 1.35	9.40 ± 1.62	10	10
Levi et al.	40/34	20/47	51.2 ± 9.9	55.1 ± 9.3	42.8 ± 5.5	44.6 ± 5.5	4.7 ± 3.8	10.3 ± 9.4	148.9 ± 50.1	172.5 ± 57.1	Unknown	Unknown	7.2 ± 1.4	8.0 ± 1.8	17	37
Schauer et al.	38/120	10/23	47.8 ± 8.6	48.2 ± 8.5	50 ± 8.4	51 ± 8.6	4.1 ± 5.1	10.7 ± 6.8	183 ± 7.6	189 ± 7.8	Unknown	Unknown	8.1 ± 2.0	8.8 ± 1.8	36	20
Nannipieri et al.	Unknown	Unknown	54 ± 8	53 ± 8	45.7 ± 5.3	46.5 ± 6.0	8.9 ± 6.0	10.1 ± 5.7	149 ± 35	170 ± 45	Unknown	Unknown	7.9 ± 1.9	8.9 ± 2.3	Unknown	Unknown
Hayes et al.	37/70	8/12	48 ± 10	51 ± 8	47 ± 10	41 ± 6	3.68 ± 4.41	9.31 ± 4.62	154.08 ± 62.46	181.98 ± 52.74	4.17 ± 1.69	3.03 ± 2.23	7.35 ± 1.45	9.78 ± 1.52	Unknown	Unknown
Kim et al.	28/128	17/46	45.9 ± 9.2	49.2 ± 8.9	50.2 ± 8.5	50.8 ± 9.1	4.8 ± 4.4	13.2 ± 7.1	141.8 ± 48.5	180.0 ± 65.1	Unknown	Unknown	7.3 ± 1.4	8.3 ± 1.6	37	49
DiGiorgi et al.	Unknown	Unknown	44.4 ± 9.7	47.3 ± 9.4	51.6 ± 10.0	51.0 ± 5.8	3.3 ± 2.5	17.3 ± 4.3	163 ± 74	177 ± 73	Unknown	Unknown	6.7 ± 1.3	8.0 ± 1.3	3	13
Torquati et al.	12/60	19/6	44.0 ± 8.9	46.4 ± 6.1	48.5 ± 7.5	51.7 ± 6.9	3.5 ± 2.8	4.3 ± 3.9	Unknown	Unknown	Unknown	Unknown	7.5 ± 1.3	8.6 ± 1.7	10	17
Yan et al.	50/29	Improved, 4/5; unaffected, 5/6	45.9 ± 10.0	Improved, 51.8 ± 10.3; unaffected, 49.4 ± 11.2	26.9 ± 4.0	Improved, 23 ± 3.6; unaffected, 23.7 ± 3.2	Unknown	Unknown	189 ± 54	Improved, 198 ± 66.6; unaffected, 201.6 ± 72	2.1 ± 0.8	Improved, 1.3 ± 0.5; unaffected, 1.6 ± 0.6	8.9 ± 1.6	Improved, 9.8 ± 2.7; unaffected, 9.7 ± 0.8	Unknown	Unknown
Jimenez et al.	3/3	2/4	Unknown	Unknown	Unknown	Unknown	Unknown	Unknown	Unknown	Unknown	Unknown	Unknown	Unknown	Unknown	Unknown	Unknown
Blackstone et al.	Unknown	Unknown	Unknown	Unknown	Unknown	Unknown	Oral medication only, 3.5 ± 4.0; oral medication and insulin, 4.7 ± 4.0	Oral medication only, 6.3 ± 5.4; oral medication and insulin, 12.8 ± 7.3	Unknown	Unknown	Unknown	Unknown	Unknown	Unknown	18	115
Biro et al.	9/8	6/16	55.6 ± 6.1	55.5 ± 8.6	45.1 ± 2.7	50.7 ± 3.5	13 ± 4.7	15.5 ± 4	Unknown	Unknown	Unknown	Unknown	7.5 ± 0.7	7.7 ± 0.6	Unknown	Unknown

All data are reported as mean ± standard deviation. Other abbreviations are as in Table 1

*FPG* fasting glucose, *HbA1c* glycosylated hemoglobin

### Quantitative Synthesis Meta-Analysis

#### BMI

A total of 1,149 T2DM patients in 13 studies were included in the meta-analysis to identify the correlation between preoperative BMI and T2DM remission [8–14, 16, 17, 19–22]. Of these, seven studies were conducted in North America, one in South America, three in Asia, one in Oceania, and one in Europe. Our meta-analysis showed no significant association between baseline BMI and T2DM remission (random model, OR = 0.45, 95 % CI = 1.40 to 2.31, *P* = 0.63). Stratified analysis demonstrated significant differences associated with Asian ethnicity (random model, OR = 2.09, 95 % CI = 1.06 to 3.13, *P* < 0.01), although there was still no statistical significance between baseline BMI and non-Asian ethnicity. Thus, Asian T2DM patients with higher baseline BMI scores may have a higher propensity for postoperative remission.

#### Patient Age

A total of 1,149 T2DM patients in 13 studies were included in the meta-analysis of the relationship between preoperative age and T2DM remission [8–14, 16, 17, 19–22]. Of these, seven studies were conducted in North America, one in South America, three in Asia, one in Oceania, and one in Europe. The results of the meta-analysis showed a negatively significant association between baseline age and T2DM remission (random model, OR = −2.46, 95 % CI = −3.90 to −1.02, *P* < 0.01), even with stratified analysis by ethnicity. No publication bias was detected by funnel plot analysis (data not shown). Thus, younger T2DM patients may have a higher remission rate.

#### Duration of Diabetes

A total of 1,555 T2DM patients in 13 studies were included in the meta-analysis of the relationship between diabetes duration and T2DM remission [8–14, 16–21]. Of these, seven studies were conducted in North America, two in Asia, one in Oceania, and three in Europe. The meta-analysis results showed a significant association between diabetes duration and T2DM remission (random model, OR = −5.22, 95 % CI = −7.39 to −3.42, *P* < 0.01), even with stratified analysis by ethnicity. No publication bias was detected by funnel plot analysis (data not shown). Thus, T2DM patients with short diabetes duration may have a higher remission rate.

#### Fasting Glucose Levels

A total of 914 T2DM patients in nine studies were included in the meta-analysis of the relationship between preoperative fasting glucose levels and T2DM remission [8–12, 14, 16, 19, 22]. Of these, three studies were conducted in North America, three in Asia, one in Oceania, and two in Europe. The meta-analysis results showed a significant association between baseline fasting glucose levels and T2DM remission (random model, OR = −19.95, 95 % CI = −31.85 to −8.05, *P* < 0.01). However, there was no relationship between fasting glucose levels and diabetes remission after stratified analysis by ethnicity (fixed model, OR = −15.16, 95 % CI = −38.58 to 8.27, *P* = 0.20). Publication bias was detected in non-Asian studies by funnel plot analysis (data not shown). Thus, T2DM patients with increased baseline fasting glucose levels may have a lower remission rate in non-Asian patients.

#### HbA1c Levels

**Fig. 2:**
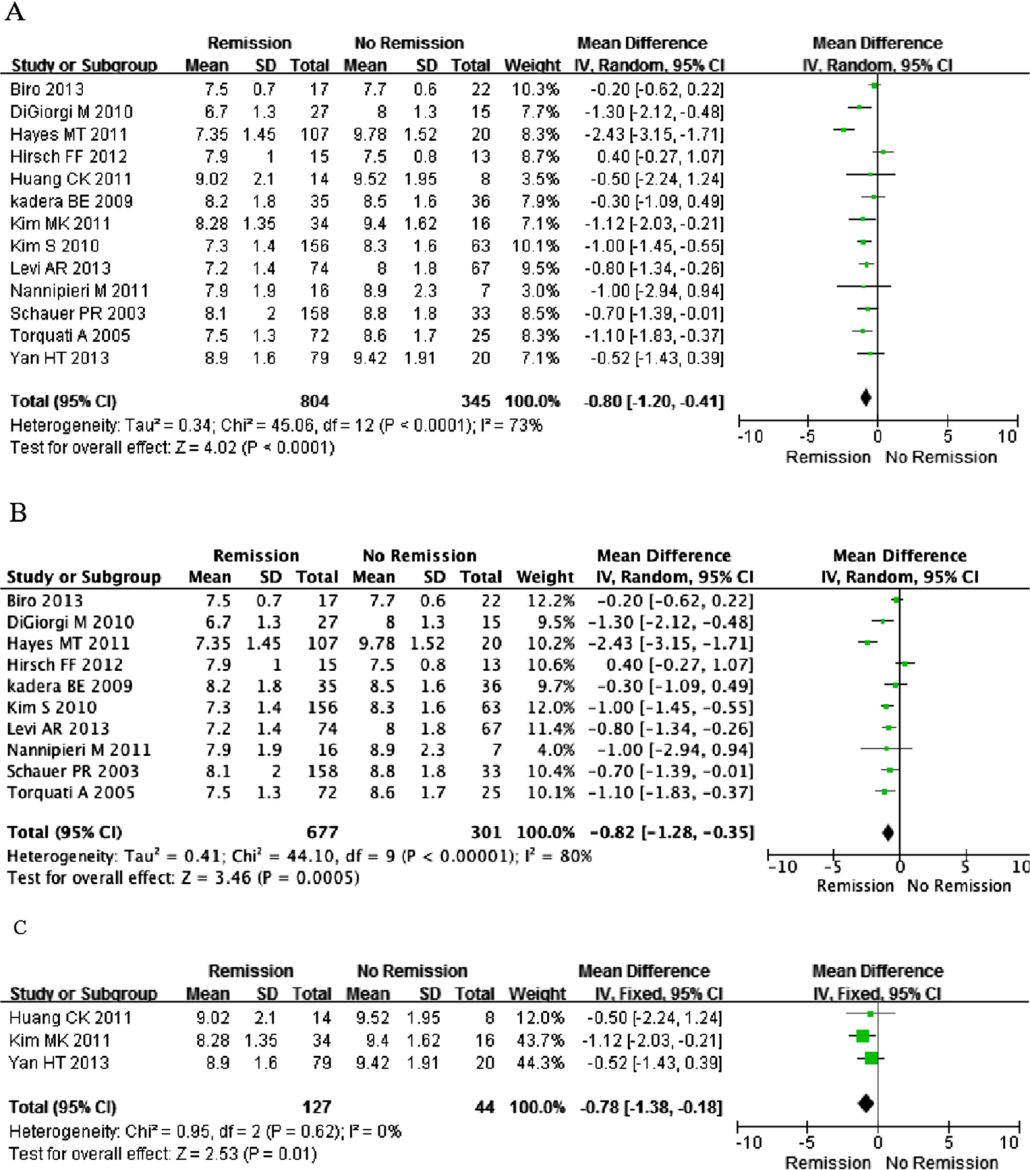
Forest plot of comparison: remission vs no remission in terms of baseline HbA1c. Mean differences are shown with 95 % CIs. **a**–**c** For HbA1c. **a** For all included studies, **b** studies from North America, **c** studies from Asian

A total of 1,149 T2DM patients in 13 studies were included in the meta-analysis of the relationship between preoperative HbA1c levels and T2DM remission [8–14, 16, 17, 19–22]. Of these, seven studies were conducted in North America, one in South America, three in Asia, one in Oceania, and one in Europe. The meta-analysis results showed a significant association between baseline HbA1c levels and T2DM remission (random model, OR = −0.80, 95 % CI = −1.20 to −0.41, *P* < 0.01) (Fig. 2), even with stratified analysis by ethnicity. No publication bias was detected by funnel plot analysis (data not shown). Thus, T2DM patients with increased baseline HbA1c levels may have a lower remission rate.

#### C-peptide Levels

A total of 149 T2DM patients in two Asian studies were included in the meta-analysis of the relationship between baseline C-peptide levels and T2DM remission [16, 22]. Our meta-analysis showed a significant association between baseline C-peptide levels and T2DM remission (random model, OR = 0.62, 95 % CI = 0.33 to 0.91, *P* < 0.01). No publication bias was detected by funnel plot analysis (data not shown). Thus, T2DM patients with higher C-peptide levels may have a higher remission rate among Asians.

#### Insulin Use

A total of 1,350 T2DM patients in 10 studies were included in the meta-analysis of the relationship between insulin use and T2DM remission [8, 9, 11, 13, 14, 16, 18–21]. Of these, six studies were conducted in the USA, two in Asia, and two in Europe. The meta-analysis results showed a significant association between insulin use and T2DM remission (random model, OR = 0.15, 95 % CI = 0.11 to 0.20, *P* < 0.01), even with stratified analysis by ethnicity. No publication bias was detected by funnel plot analysis (data not shown). Thus, non-Asian patients on insulin therapy before surgery may have a lower remission rate.

#### Gender

A total of 1,113 T2DM patients in 13 studies were included in the meta-analysis of the relationship between gender and T2DM remission [8, 10, 11, 13–17, 19–22]. Of these, seven studies were conducted in the USA, three in Asia, one in Oceania, and two in Europe. The meta-analysis results showed an insignificant association between gender and T2DM remission. No publication bias was detected by funnel plot analysis (data not shown). Thus, gender may not be a predictor of T2DM remission after bariatric surgery.

### Publication Bias and Heterogeneity Analyses

The funnel plots were inspected for underlying geometric distribution and were found to be symmetrical in most models. No publication bias was detected in the funnel plots using Begg’s method with the exception of fasting glucose levels, suggesting the absence of publication bias in most meta-analysis models. Similarly, significant heterogeneity was observed in most models. To investigate this further, we performed meta-regression analysis to identify the source of heterogeneity using the criteria of publication year, ratio of males, sample size, T2DM remission criterion, and quality scoring. Univariate meta-regression analysis revealed that none of the tested covariates could explain the observed differences in heterogeneity between the studies. Moreover, when the data were stratified by ethnicity, heterogeneity was not significantly decreased or eliminated.

### Sensitivity Analysis

Sensitivity analysis was performed to assess the influence of each individual study on the pooled OR values by sequentially repeating the meta-analysis excluding one study at a time. The results of this analysis suggested that the pooled OR values were not significantly affected (data not shown), indicating statistically robust results.

## Discussion

Complementing previous comprehensive studies, our meta-analysis provides additional results for the identification of potential predictors of diabetes remission after bariatric surgery by stratifying patients according to ethnicity. The major findings of our analysis are that for both Asian and non-Asian obese T2DM patients, older age, long history of diabetes, insulin use, and poor glycemic control can be assumed to be negative predictors for failure of diabetes remission in the postoperative course. Moreover, our results indicated that gender and baseline BMI do not play a role in diabetes remission. In addition, Asian T2DM patients appeared to have a better remission rate of diabetes after bariatric surgery, which was also correlated with lower severity of T2DM.

Previous observational studies suggested age, shorter disease duration, and appropriate glycemic control prior to surgery as predictors of disease remission [23–32]. The results of our meta-analysis further confirmed these findings. Thus, age, diabetes duration, and appropriate glycemic control appear to be selective criteria among Asian and non-Asian patients.

Furthermore, we confirmed that disease severity is an important predictive factor of the extent of improvement that can be potentially achieved after bariatric surgery, particularly for Asian patients. High level of HbA1c is widely considered as a marker for uncontrolled T2DM. It is a common practice among many surgical teams to exclude patients from surgical treatment or at least to postpone further scheduled operation due to a high level of HbA1c. Although there is no consensus regarding not operating these patients, the assumption that patients with higher HbA1c are at greater risks of perioperative morbidity and mortality is probably extrapolated from bariatric procedures. The selection of which patients should not be operated due to advanced or severe uncontrolled T2DM may be one of the reasons why the difference between the T2DM remission and non-remission group is not much larger. The results of our meta-analysis also confirmed that the higher HbA1c is negatively associated with the T2DM remission, the further a study should explore the HbA1c cutoff setting. On the other hand, previous studies have shown that relatively less deterioration of β cell function at the time of surgery may maximize the effect of surgery to increase β cell insulin secretion [28]. In contrast, a worse initial degree of β cell dysfunction may lower the chances of T2DM remission [20]. Therefore, preoperative assessment of insulin secretion capacity could be proven useful to grade the stage and severity of T2DM and to predict the diabetes status after RYGB surgery. Measurement of fasting C-peptide levels is a commonly accepted method to assess insulin secretion [33, 34], which is often used to estimate disease severity. Improvement in C-peptide levels has been confirmed to significantly reduce the probability of diabetes remission [35, 36]. The results of our meta-analysis showing that T2DM patients with higher C-peptide levels achieved a better remission rate support this viewpoint. However, there were no available data to appropriately analyze the relationship between C-peptide and T2DM remission in non-Asian patients. Therefore, further studies are needed to confirm a possible correlation among other ethnic groups.

Interestingly, our meta-analysis yielded some unexpected and provocative results regarding the association between BMI and diabetes remission. As is well known, an increase in BMI is closely related to protracted insulin resistance [37]. The main mechanism of T2DM remission after bariatric surgery is the amelioration of insulin resistance; therefore, a high BMI may hold predictive value for the success of metabolic surgery for T2DM [38–40]. However, in our meta-analysis, we found that a high BMI may be predictive of the success of metabolic surgery for T2DM only in Asian patients. Therefore, it remains uncertain whether BMI is a predictive factor in non-Asian patients. We believe that our non-significant result may have been due to the inclusion of non-Asian patients in the analyzed studies with BMI greater than the cutoff value. However, given that a lower baseline BMI is a negative predictor of T2DM remission, we should be cautious with the extrapolation of the benefits of surgery to diabetic patients who have a relatively low BMI. This finding highlights the importance of the implication of BMI for patient selection and indications for surgery among Asian patients.

Another issue regarding successful outcomes is whether the use of insulin had an adverse effect on the remission rate of T2DM. Because most patients requiring insulin therapy had a longer known T2DM duration and greater preoperative fasting glucose and HbA1c levels than those not requiring insulin, insulin treatment may impact the severity of diabetes to some degree. Compared with non-Asian patients, T2DM in Asian patients is characterized by worse deterioration of early phase insulin secretion, which appears to be associated with severe impairment of β cell function. Specifically, patients dependent on insulin therapy may have worse β cell function. Thus, insulin independence may be a predictor of better diabetes remission. Our meta-analysis further confirmed this. Therefore, diabetes remission was more unpronounced in patients requiring insulin therapy compared with prescribed oral hypoglycemic agents. These factors, although statistically independent, are also interrelated. The lower rate of durable remission in insulin-treated subjects is possibly related to a lack of sufficient residual β cell mass. Diabetes duration is known to reflect the residual β cell mass in T2DM patients, both in morbidly and non-morbidly obese patients. Older age is also associated with lower insulin sensitivity and diminished insulin secretion, and a high BMI is associated with higher C-peptide levels [2]. At the same time, HbA1c levels are an indicator of long-standing hyperglycemia, which has been demonstrated to have direct glucotoxic effects on β cells to reduce β cell secretory function [41]. Thus, HbA1c levels are also related to decreased C-peptide levels. This combination of factors indicates that patients with higher C-peptide levels are more likely to achieve remission. Of note in this regard, β cell function may be a predictor of T2DM. Therefore, it is possible that C-peptide levels are the most important underlying clinical denominator for T2DM remission.

There were some limitations of this meta-analysis. First, our review was limited by the studies using different criteria to define T2DM remission. To address this issue, we repeated the analysis by limiting our study to 13 articles that used diabetes remission criteria equal to or stricter than the ADA criteria; we still observed consistent results. Second, all the included studies were relatively short-term, with durations of 6 to 36 months. Third, a high degree of heterogeneity existed in most analyses.

Despite these shortcomings, our analysis of the included studies suggested that an improved remission rate can be expected in patients characterized by younger age, short diabetes duration, better glycemic control, and better β cell function. The results presented here shed new light on the importance of preoperative patient selection. Nonetheless, further randomized studies to compare the reported data of predictive factors using the ADA criteria to define T2DM remission with adequate long-term follow-up among different populations are warranted to establish appropriate selection criteria.
